# Crystal Structure of *Schistosoma mansoni* Adenosine Phosphorylase/5’-Methylthioadenosine Phosphorylase and Its Importance on Adenosine Salvage Pathway

**DOI:** 10.1371/journal.pntd.0005178

**Published:** 2016-12-09

**Authors:** Juliana Roberta Torini, José Brandão-Neto, Ricardo DeMarco, Humberto D'Muniz Pereira

**Affiliations:** 1 Laboratório de Biologia Estrutural, Instituto de Física de São Carlos, Universidade de São Paulo, São Carlos, SP, Brazil; 2 Diamond Light Source. Harwell Science and Innovation Campus, Didcot, Oxfordshire, United Kingdom; Institut Pasteur de Montevideo, URUGUAY

## Abstract

*Schistosoma mansoni* do not have *de novo* purine pathways and rely on purine salvage for their purine supply. It has been demonstrated that, unlike humans, the *S*. *mansoni* is able to produce adenine directly from adenosine, although the enzyme responsible for this activity was unknown. In the present work we show that *S*. *mansoni* 5´-deoxy-5´-methylthioadenosine phosphorylase (MTAP, E.C. 2.4.2.28) is capable of use adenosine as a substrate to the production of adenine. Through kinetics assays, we show that the *Schistosoma mansoni* MTAP (*Sm*MTAP), unlike the mammalian MTAP, uses adenosine substrate with the same efficiency as MTA phosphorolysis, which suggests that this enzyme is part of the purine pathway salvage in *S*. *mansoni* and could be a promising target for anti-schistosoma therapies. Here, we present 13 *Sm*MTAP structures from the wild type (WT), including three single and one double mutant, and generate a solid structural framework for structure description. These crystal structures of *Sm*MTAP reveal that the active site contains three substitutions within and near the active site when compared to it mammalian counterpart, thus opening up the possibility of developing specific inhibitors to the parasite MTAP. The structural and kinetic data for 5 substrates reveal the structural basis for this interaction, providing substract for inteligent design of new compounds for block this enzyme activity.

## Author Summary

The huge challenge in parasitic chemotherapy development is finding a specific compound to attack the parasite organisms without damaging their host. *Schistosoma mansoni*, which is the causative agent of schistosomiasis, is one of the major health concerns in the developing world. Purine bases are essential for organisms that make DNA, RNA and energetic molecules during parasitic growth and egg laying. The parasites depend entirely on re-utilizing existing purines, not being able to synthesize them from more simple molecules. The adenosine phosphorylase is an important activity for this process and kinetic assays we performed with this *S. mansoni* MTAP confirm that it displays this specific activity that is not present in the human metabolism. Therefore, understanding the properties of this enzyme is an important step in achieving an efficient anti-schistosomiasis drug with minimal collateral effects to humans.

## Introduction

The purine metabolism in the human parasite *Schistosoma mansoni* was extensively studied in the 1970s and 1980s by Senft and collaborators [1–8] and Dovey [9]. These studies demonstrated that adults and schistosomules of *S*. *mansoni* do not have the capacity to synthesize purine nucleotides *de novo*, instead depending solely on the purine salvage pathway to meet their purine requirements.

One striking difference between *S*. *mansoni* and the human host (and also from other mammals) is their phosphorolytic capacity to cleave adenosine into adenine [4]. The presence of enzymatic activity for adenosine phosphorylase (AP) (EC None), yielding adenine and ribose-1-phosphate (S1 Fig), was detected by Senft *et al*. [1] in both extracts and vomitus of *S*. *mansoni*. The free adenine base released in this reaction is utilized by adenine phosphoribosyltransferase (APRT) (EC 2.4.2.7) to generate AMP, which in turn is converted into ADP and further into ATP via the action of adenylate kinase (ADK) (EC 2.7.4.3) and nucleoside diphosphate kinase (NDPK) (EC 2.7.4.6) enzymes.

When cell-free extracts of *S*. *mansoni* were incubated with [^14^C]-adenosine in the presence of phosphoribosyl pyrophosphate (PRPP), approximately 70% of the initial radioactivity was detected as nucleotides after 120 minutes. In contrast, when PRPP was omitted, negligible amounts of radioactivity were presented as nucleotides, even after 120 minutes of incubation [1]. When the worms were incubated with [^14^C]-adenine, the rapid formation of nucleotides was observed. These results were confirmed by Dovey *et al*. [9], who demonstrated that schistosomules also have a significant adenosine phosphorylase activity (1.12 nmol min^-1^ mg^-1^ protein); this activity was highest in schistosomule extracts. The AP activity was inhibited by adenosine analogue formycin A (75% inhibition at 1 mM). *S*. *mansoni* also exhibits high levels of APRT activity (2.32 ± 0.42 nmol min -1 mg -1 protein); thus, adenosine phosphorylase-APRT chain reactions must be the main route for adenosine incorporation. This fact correlates with the relative abundance of adenosine in the serum of the mammalian host (0.2–5.0 μM) hmdb.ca [9].

The use of adenosine analogues alone or in combination with nucleoside transport inhibitors has been employed both *in vitro* and *in vivo* [10–14]. One of these analogues, tubercidin (7-deazadenosine), is incorporated at the nucleotide level and inhibits *Schistosoma* motility with an IC50 of 1 μM, and Formycin A (8-aza-9-deazaadenosine) inhibits 73% of the adenosine phosphorylase activity at 1 mM [15]. These examples indicate the feasibility of using purine analogues as a scaffold for the development of new antiparasitic agents.

Because the *S*. *mansoni* genome does not codify an adenosine phosphorylase, one alternative to fulfilling this activity is 5´-deoxy-5´-methylthioadenosine phosphorylase (E.C. 2.4.2.28) (MTAP). This enzyme catalyzes the reversible phosphorolysis of 5´-deoxy-5´-methylthioadenosine (MTA) to free adenine and 5´-deoxy-5´-methythioribose-1-phosphate (MTR1P) (S2 Fig). In humans, the MTAP participates mainly in the polyamine pathway [16]. The MTAP enzyme is present in some parasites (*Leishmainia donovani*, *S*. *mansoni*, *Trypanosoma cruzi* and *brucei*) and absent in others (*Giardia lamblia*, *Plasmodium falciparum* and *entamoeba invadens*). When it is present, parasite MTAP is similar to the mammalian MTAP, although it does differ in some characteristics: it has a low K_M_ for the adenosine and some of their analogues (2´deoxy or 2´,3´dideoxyribose analogues) [17].

Here, we describe the structure and kinetics parameters of a *S*. *mansoni* MTAP (*Sm*MTAP) and its active site mutants. By analyzing both sets of data, we can hypothesize that MTAP and AP, as described above in a series of articles, are the same entity in worms. Furthermore, a comparison of the K_M_ levels reveals that *S*. *mansoni* MTAP can use adenosine with the same efficiency as MTA, which is one fundamental difference between *S*. *mansoni* and its host and reveals a potential target for schistosomiasis drug development.

## Materials and Methods

### Bacterial strains, plasmids and chemical reagents

The pET28a expression system, *E*. *coli* BL21(DE3) strains was purchased from Novagen. The cloning vector pTZ57R/T and Taq DNA Polymerase were purchased from Thermo Fisher Scientific. The Wizard *Plus* SV Minipreps DNA Purification System was purchased from Promega, and restriction enzymes were purchased from New England BioLabs. The xanthine oxidase from bovine milk and all nucleosides were purchased from Sigma-Aldrich.

### Cloning, expression and purification of recombinant *Sm*MTAP

From the total mRNA from the adult worm, strand cDNAs were synthesized by RT-PCR employing the SuperScript III First-Strand Synthesis System from Promega. Forward (5’-CTG*GCTAGC*ATGTCTAAAGTTAAGGTTGGAATTATTG-3’) and reverse (5’- CTG*CTCGAG*CCAATTTACTTCATGTTTATTTGTCATTAC-3’) primers containing *NheI* and *XhoI* restriction sites (in italics) were designed for subcloning into the pET28a expression vector. The RT product was used as a template for PCR; after adenylation, the amplification product was cloned into the pTZ57R/T vector and transformed into *Escherichia coli* DH5α cells. Transformants were selected using the chromogenic substrate X-gal and by colony PCR. The MTAP gene was digested with *NheI* and *XhoI* (New England Biolabs) and recovered on a 1% agarose gel using the Promega Wizard SV Gel and PCR Clean Up kit. The pET28a-MTAP construct was synthesized by treatment with T4 DNA ligase (New England Biolabs) using pre-digested pET28a vector with the same enzymes. The fusion plasmid was used in the transformation of *Escherichia coli* BL21(DE3) cells. The transformed *E*. *coli* was confirmed by PCR colony. Protein expression was performed in 1 L 2xYT medium in the presence of 50 μg/mL kanamycin, inoculated with an overnight culture. The cells were incubated at 37°C to an OD600 of approximately 0.6 and were induced with 0.1 mM of isopropyl β-D-thiogalactopyranoside (IPTG) for 4 h. The cells were harvested by centrifugation at 6000 g for 45 minutes at 4°C and lysed by sonication in 50 mM NaH_2_PO_4_, pH 7.4, 300 mM NaCl, 10 mM imidazol and 5 mM β-mercaptoethanol. The lysate was clarified by centrifugation at 9,000 g for 20 minutes at 4°C. The soluble fraction was applied on a Co-NTA agarose column (Clontech) and washed with 50 mM NaH_2_PO_4_ pH 7.4, 300 mM NaCl, 20 mM imidazole and 5 mM β-mercaptoethanol. The protein was eluted with 50 mM NaH_2_PO_4_, pH 7.4, 300 mM NaCl, 200 mM imidazole and 5 mM β-mercaptoethanol. The enzyme was dialyzed against 20 mM Tris, pH 7.4, 200 mM NaCl and 10 mM β-mercaptoethanol. All stages of *Sm*MTAP production were visualized by SDS-PAGE.

The single *Sm*MTAP mutants (S12T, N87T and Q289L) were prepared by Mutagenex (Suwanee-USA), and the double (S12T/N87T) and triple mutants (S12T/N87T/Q289L) were prepared by Cellco Biotech (São Carlos-Brazil). The protocols for the expression and purification of the mutants were the same as those used for wild-type *Sm*MTAP.

### *Sm*MTAP kinetic assays

The kinetic parameters for adenosine, MTA, 2-deoxyadenosine and phosphate (PO_4_) were measured by coupled assay by xanthine oxidase [18]. In this method, the xanthine oxidase converts free adenine from MTA or Ado into 2,8-dihydroxyadenine, resulting in an increase in the absorbance at A305 nm (ε = 15.500 AU [19]). Kinetic parameters were calculated in sextuplicate at room temperature in a 200 μL reaction mix containing 100 mM potassium phosphate buffer at pH 7.4, an adequate quantity of substrate and 0.3 units of xanthine oxidase from bovine milk (Sigma-Aldrich). The reaction was started by adding 50 nM of *Sm*MTAP to the reaction mixture, and the OD305 was immediately monitored using a SPECTRAmax PLUS384 spectrophotometer (Molecular Devices, USA). For PO_4_ kinetics 100 mM Hepes pH 7.4 was used. The kinetic parameters (K_M_ and k_cat_) were derived from non-linear least-squares fits of the Michaelis-Menten equation in the Graphpad Prism software using the experimental data.

### Crystallization, X-ray data collection and processing

The initial crystallization conditions for *Sm*MTAP were determined by a Honeybee 961 robot (Genomic Solutions) using crystallization screen solutions from Hampton Research and Qiagen, where several conditions yielded crystals of varying quality. These conditions were optimized by varying the pH and PEG concentration, which resulted in diffraction-quality crystals that grew at 18°C using the hanging-drop technique. The best conditions were 100 mM Bis-tris or MES buffer, pH 6.1–6.7 and 14–18% PEG 3350 with 6 μl drops containing a 1:1 mixture of protein (6 mg/mL)/crystallization solution, incubated in a 500 μL reservoir solution. The same procedure was employed for single, double and triple mutants. The triple mutants did not generate crystals, even in a new crystallization screening, due to solubility problems.

The *Sm*MTAP wild-type and mutant crystals were also grown in the presence of the ligands (5 mM) MTA, adenosine, ribose 1-phosphate and tubercidin. The crystals were mounted in a nylon-fiber loop, cryoprotected with 20% glycerol or PEG200 in the mother solution and cooled in liquid nitrogen.

Diffraction data were measured using synchrotron radiation on a Beamline MX2 of the Laboratório Nacional de Luz Síncrotron (LNLS, Campinas, Brazil) and Beamlines I02, I04 and I04-1 at Diamond Light Source (DLS, Harwell, UK). The data were indexed, integrated and scaled using the MOSFLM/SCALA programs [20,21] to the data from LNLS, and the Xia2 [22] and XDS [23] programs to the data from DLS.

### Structure solution and refinement

The *Sm*MTAP-apo enzyme structure was solved by molecular replacement using Phaser [24], employing the human MTAP monomer (PDB ID 1CB0 [25]) as a search model because it shares 47% of sequence identity. The remaining wild-type and mutant structures were also solved by molecular replacement, using one of the previously refined structures as a model. The refinement was carried out using Phenix [26], and the model was constructed using COOT [27] with weighted 2Fo–Fc and Fo–Fc electron density maps. The full statistics of data collection and refinement are shown in Table 1. In all cases, the behavior of R_Free_ was used as the principal criterion for validating the refinement protocol, and the stereochemical quality of the model was evaluated with Molprobity [28]. The collection, processing and refinement are shown in Table 1. The coordinates and structure factors have been deposited with the PDB under the following codes: *Sm*MTAP-tubercidin 4L5A; *Sm*MTAP-adenine in space group *P212121* 4L5C; *Sm*MTAP-APO 4L5Y; *Sm*MTAP-adenine 4L6I; *Sm*MTAP-S12T-APO 5F73; *Sm*MTAP-S12T-adenine 5F77; *Sm*MTAP-S12T-MTA 5F76; *Sm*MTAP-N87T-APO 5F78; *Sm*MTAP- N87T- adenine 5F7J; *Sm*MTAP-Q289L-APO 5F70; *Sm*MTAP- Q289L-Tubercidin 5F7X; *Sm*MTAP-S12T-N87T APO 5F7Z; and *Sm*MTAP- S12T-N87T adenine 5FAK.

**Table 1:** Data collection, processing and refinement statistics.

	*Sm*MTAP-apo	*Sm*MTAP-ade	*Sm*MTAP-tub	*Sm*MTAP-ade-gol	S12Tapo	S12T-Ade	S12T-MTA	N87T-Apo	N87T-Ade	Q289L-Ade	Q289L-Tub	2M-apo	2M-Ade
Detector	MarMosaic 225	MarMosaic 225	Quantum 315r	Quantum 315r	Pilatus 2M	Pilatus 2M	Pilatus 2M	Pilatus 2M	Pilatus 6MF	Pilatus 2M	Pilatus 2M	Pilatus 6MF	Pilatus 6MF
Cell parameters (Å) a; b;c α; β; γ	80.63; 82.40; 150.28 90.0; 101.6; 90.0	81.27; 82.55; 150.30 90.0; 100.6; 90.0	81.07; 82.68; 150.56 90;101.34;90	102.58; 135.71;145.05 90.0; 90.0; 90.0	81.44;82.16;150.91 90; 101.5;90	74.46; 84.65;75.9290; 106.28; 90	76.81; 73.91; 81.29 90; 102.44; 90	81.19; 82.23; 150.7590; 101.55; 90	81.49; 82.11; 150.27 90; 101.10; 90	74.35; 81.87; 81.7690; 101.95;90	74.36; 81.9; 81.7390; 102.16; 90	80.70; 82.26; 150.05 90; 101.42; 90	81.06; 82.09; 150.15 90; 101.23; 90
Space Group	P2_1_	P2_1_	P2_1_	P 2_1_ 2_1_ 2_1_	P 2_1_	P 2_1_	P 2_1_	P 2_1_	P 2_1_	P 2_1_	P 2_1_	P 2_1_	P 2_1_
Resolution (Å)	20.0–2.1 (2.22–2.10)	30.0–2.1(2.21–2.10)	30.0–2.30 (2.44–2.30)	59.22–2.08 (2.13–1.08)	39.58–2.06 (2.11–2.06)	72.88–2.02 (2.07–2.02)	49.46–1.95 (2.00–1.95)	28.05–1.85 (1.95–1.85)	76.76–1.66 (1.72–1.66)	27.19–1.81 (1.91–1.81)	48.89–1.77 (1.82–1.77)	82.26–1.8 (1.94–1.80)	79.51–1.87 (1.92–1.97)
X ray Source	W01B-MX2	W01B-MX2	I02	I02	I04-1	I04-1	I04-1	I04-1	I04	I04-1	I04-1	I04	I04
λ (Å)	1.45	1.43	0.9686	0.9795	0.92	0.92	0.92	0.92	0.9795	0.92	0.92	0.9795	0.9795
Multiplicity	4.1 (3.9)	4.9 (5,1)	1.94 (1.92)	7.2 (7.3)	4.2 (3.8)	4.2 (4.2)	3.9 (4.0)	5.4 (5.3)	3.8 (2.3)	3.8 (3.8)	3.8 (3.7)	3.3 (3.3)	4.3 (4.4)
R_merge (%)_	10.2 (62.6)	12.1 (51.1)	4.8 (23.6)	11.9 (69.5)	9.4 (59.5)	8.6 (61.5)	8.9 (52.2)	8.9 (56.6)	7.3 (65.0)	10.8 (47.3)	6.8 (48.7)	8.6 (67.0)	8.1 (92.9)
R_pim_ (%)	N.A	6.1 (24.6)	N.A	5.4 (30.6)	5.2 (34.6)	7.3 (52.0)	0.79 (46.2)	6.3 (40.7)	4.7 (53.9)	9.7 (42.6)	6.0 (42.9)	5.6 (43.6)	5.7 (59.1)
CC(1/2)	N.A	N.A	99.7 (90.7)	N.A	0.99 (0.77)	0.99 (0.79)	0.99 (0.77)	0.99 (0.60)	0.99 (0.47)	0.98 (0.75)	0.99 (0.75)	0.99 (0.74)	0.99 (0.53)
Completeness (%)	98.4 (95.9)	91.4 (100)	93.2 (92.5)	99.8 (99.6)	99.8 (94.3)	99.2 (98.2–99.1)	96.8 (96.9)	97.5 (97.7)	99.5 (75.7)	96.7 (97.8)	97.3 (89.6)	99.9 (99.9)	97.7 (96.6)
Reflections	459122 (67754)	514601 (15929)	308613 (48737)	880364 (10016)	449950 (31460)	249705 (18323)	244597 (18537)	874609 (124104)	838202 (42414)	318481 (46630)	348470 (22508)	593295 (111133)	176595 (49849)
Unique Reflections	111617 (17346)	104402 (3645)	158912 (25362)	122620 (1528)	119129 (8332)	58891 (4347)	62658 (4658)	161035 (23481)	221276 (18113)	83708 (12303)	90698 (6156)	178037 (36498)	155607 (11378)
*I*/σ	10.16 (2.35)	8.3 (3.0)	13.02 (3.53)	9.8 (3.00)	11.2 (2.4)	10.1 (2.2)	9.3 (2.2)	11.0 (2.8)	7.8 (1.1)	6.5 (2.1)	10.4 (2.1)	7.1 (1.6)	10.3 (1.3)
Reflections used for Refinement	111591	103947	158769	122450	120787	58868	62633	160675	218591	83329	90672	177099	155532
R(%)**	18.54	19.23	18.97	19.26	17.53	16.92	16.63	17.12	19.46	18.21	16.37	21.07	18.11
R_free_(%)**	22.66	24.10	21.91	22.18	21.62	21.51	19.40	19.95	22.67	21.11	19.23	23.54	21.47
N° of protein atoms	12650	13058	12866	13260	12769	6544	6568	13039	13082	6489	6483	12797	12921
N° of ligant atoms	30	60	142	96	30	45	75	30	55	15	72	30	70
B (Å^2^)	26.38	24.78	28.32	24.91	25.92	26.14	21.98	17.36	19.42	15.69	20.19	21.79	26.43
Coordinate Error (ML based) (Å)	0.25	0.24	0.24	0.23	0.23	0.25	0.18	0.17	0.23	0.18	0.17	0.27	0.23
Phase error (°)	23.69	26.38	22.58	27.14	23.40	23.30	19.61	19.99	28.0	22.14	19.44	28.10	24.53
*Ramachandran Plot*	* *	* *	* *	* *	* *	* *	* *	* *	* *	* *	* *	* *	* *
Favored (%)	98.98	98.65	98.86	98.43	98.92	89.47	99.53	99.0	98.94	98.82	98.81	98.63	99.17
Allowed (%)	1.02	1.75	1.14	1.57	0.96	1.53	0.47	1.0	1.0	1.07	0.95	1.25	0.83
Outliers (%)	0.00	0	0.00	0.00	0.12	0.0	0.0	0.0	0.06	0.12	0.24	0.12	0.0
All-atom Clashscore	1.96	3.79	2.67	2.40	1.93	2.82	1.13	3.08	2.44	4.02	1.91	3.11	2.94
*RMSD from ideal geometry*	* *	* *	* *	* *	* *	* *	* *	* *	* *	* *	* *	* *	* *
r.m.s. bond lengths (Å)	0.004	0.007	0.003	0.003	0.007	0.004	0.006	0.003	0.008	0.004	0.006	0.002	0.005
r.m.s. bond angles (°)	0.792	1078	0.791	0.683	0.796	0.618	0.849	0.561	0.895	0.656	0.859	0.532	0.742
*PDB ID*	4L5Y	4L6I	4L5A	4L5C	5F73	5F77	5F76	5F78	5F7J	5F7O	5F7X	5F7Z	5FAK

## Results

### *Sm*MTAP sequence analysis, expression and purification

The gene model Smp_028190, which was derived from the annotation of the *S*. *mansoni* genome [29], was selected as a putative methylthioadenosine phosphorylase due to the presence of a conserved MTAP domain (TIGR01694- evalue e-99). Alignment of the nucleotide sequence of this model with *S*. *mansoni* transcript sequences from dbEST using blastn programs resulted in only the first 844 bases of this putative transcript of 888 bases being aligned to the transcript sequences. The 44 bases at the 3’ end of this model correspond to an exon predicted on bases 37729–37685 of the W chromosome and did not align to any transcript, which probably represents an error in the genome annotation. Indeed, the mapping of two ESTs that were partially aligned to this model (Accesion number EX708725.1 and EX708663.1) into the genome suggest a different 3’ end exon on bases 46092–46194 of the W chromosome. We designed primers for the amplification of the full-length coding region of MTAP based on the 5’ end from the gene model and on this new 3’ end and were able to amplify a ~900 bp amplicon. Sequencing of this amplicon confirmed this corrected gene model based on EST evidence. This new transcript sequence was deposited on NCBI under accession number JQ071534.1.

**Fig 1:**
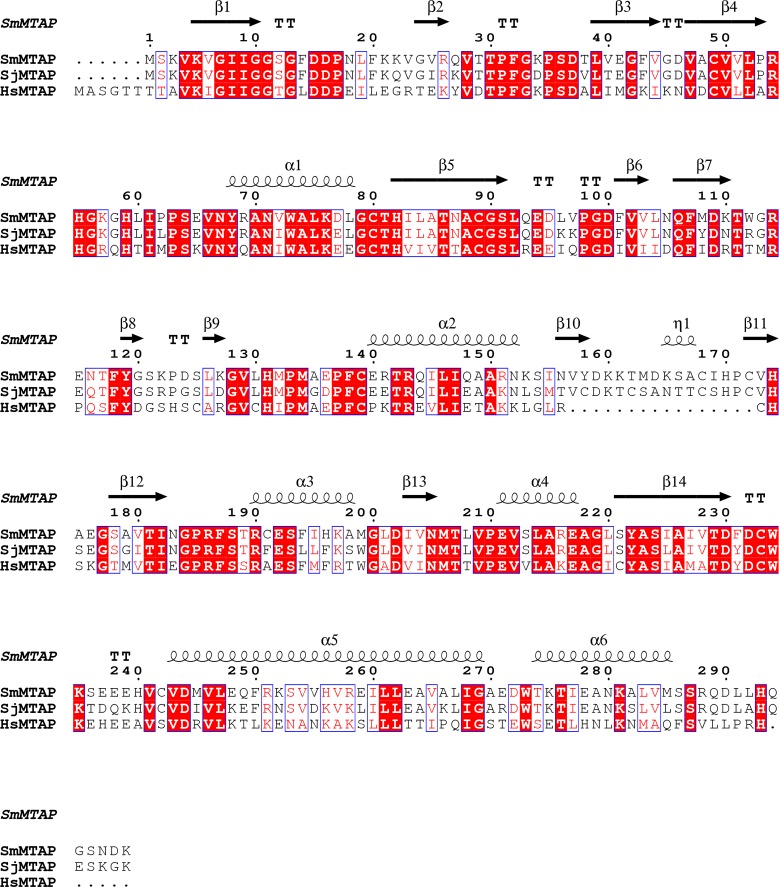
Sequence alignment of MTAPs from *S*. *mansoni* (*Sm*MTAP), *S. japonicum* (*Sj*MTAP) and human (*Hs*MTAP). *Sm*MTAP shares 77% and 47% identity with *Sj*MTAP and *Hs*MTAP, respectively. The *Sm*MTAP secondary structure elements are labeled and shown as arrows and helices. One sequence insertion also present in both *Schistosoma* species could be observed between beta strands 10 and 11.

The *Sm*MTAP gene codes a protein with 299 amino acids and an expected molecular weight of 32.913.9 Da. *Sm*MTAP shares 77% and 47% sequence identity with *S*. *japonicum* and human MTAP, respectively (Fig 1). The sequences from the two schistosomes have a 16-amino-acid insertion between beta strands 10 and 11. This insertion does not have high sequence identity between schistosome MTAPs, sharing 43.7% identity versus 77% identity for the full sequence, respectively.

After analyzing both alignments and the *Sm*MTAP structure (as discussed in detail below), we found three substitutions in the active site compared to the human MTAP. To characterize the structural differences between human and *S*. *mansoni* MTAPs active sites and the *Sm*MTAP kinetic properties, we prepared three single mutants (S12T, N87T and Q289L), a double mutant (S12T/N87T) and a triple mutant (S12T/N87T/Q289L). We obtained the structures of the single mutants and the double mutant. The triple mutant protein was highly insoluble and did not crystallize in *Sm*MTAP crystallization conditions or produce crystals, even after a new crystallization screening.

Recombinant *Sm*MTAP protein was successfully expressed using pET28a vector and purified in a single step using a cobalt affinity column, yielding ~120 mg per liter of 2xYT medium. *Sm*MTAP expression and purification were visualized by SDS PAGE gel (data not shown). The subunit molecular weight of the expressed and tagged protein was calculated to be 33.9 kDa, as observed by denaturing gel electrophoresis after purification.

### *Sm*MTAP structure description

**Fig 2:**
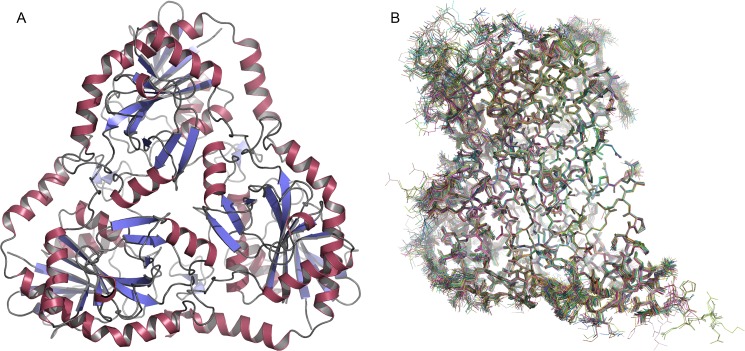
A. *Sm*MTAP trimeric structure in cartoon scheme. Beta strands are colored blue and helices in red. B. Superposition of 66 monomers of 22 independent trimers from 13 different *Sm*MTAP structures. The central beta sheet core is the invariant part of the structure. Gate loop suffers high conformational plasticity.

Crystallization and X-ray diffraction of *Sm*MTAP crystals allowed the resolution of 13 different structures, including different ligands (adenine, MTA, tubercidin and sulphate) of the WT and mutated *Sm*MTAP. With the exception of one orthorhombic structure (*P*2_1_2_1_2_1_) (in more than 100 datasets collected), *Sm*MTAP crystallized in the monoclinic space group P2_1_ with one or two trimers to an asymmetric unit. A comparison of structures from *Sm*MTAP derived from 22 independent trimers observed in 13 different conditions (Fig 2A) allows a comprehensive description of the protein structure. The observed overall fold of *Sm*MTAP is similar to that of members from NP-1 family of low-molecular weight purine nucleoside phosphorylases (PNP), which consist of a central β-sheet distorted barrel surrounded by α-helices (Fig 2A), as observed in other MTAPs from human and bacteria.

Superimposition of the 66 monomers composing the studied trimers resulted in a solid structural framework, thus allowing the detection of several conformational modifications (Fig 2B) that were related to the binding of different ligands and mutations.

**Fig 3:**
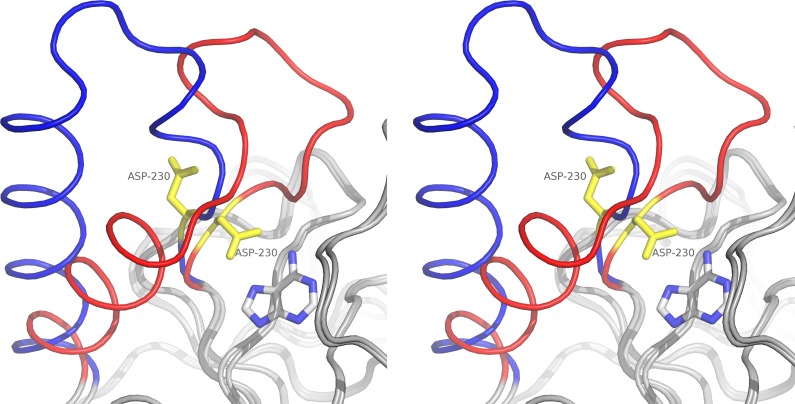
Stereo image of gate loop movement between adenine complex structure (red) and APO (blue). The D230 main residue in the active site is shown in yellow. The movement involves residues 229–253. In the APO structure, D230 points away from the active site. The presence of the base or base moiety in the base bind site appears to be necessary to "close" the gate loop in the base interacting conformation.

The trimers and monomers are similar to within an RMSd of 0.23 and 0.22 Å, respectively, and the regions comprising residues 9–21 (phosphate loop), 119–128, 159–169 and 229–254 (gate loop) are the most flexible parts of the *Sm*MTAP and undergo large conformational changes induced by ligand binding (Fig 3).

The *Sm*MTAP-adenine complex is the most common structure obtained (23 of the 66 monomers). Several structures from co-crystallization experiments with adenosine or MTA show adenine in the active site, even in the expected "apo" state. Further, adenine was identified in the *Sm*MTAP active site after crystallization and structural solution and was probably acquired from the bacterial source.

The Base Binding Sites (BBS) of two of the six *Sm*MTAP-adenine complexes obtained (*Sm*MTAP *P*2_1_2_1_2_1_ and S12T mutant) were fully occupied by adenine. In other complexes, adenine was present in only two subunits of the trimer. The subunits without adenine preferentially assume the conformation of the APO structure (open conformation) of the gate loop (residues 231–242) and part of the helix 5 (residues 240–252). Indeed, this part of helix 5 assumes two different conformations in APO and bounded structures. These conformational changes mainly involve residues 233–241.

In 20 of the 30 monomers that are bound to adenine, the gate loop is in the closed conformation, which indicates that adenine binding could promote gate loop ordering. However, it is intriguing that subunit E of the double-mutant (S12T/N87T) and subunits B and C of the S12T mutant were found in the open conformation, even when they were bound to adenine.

**Fig 4:**
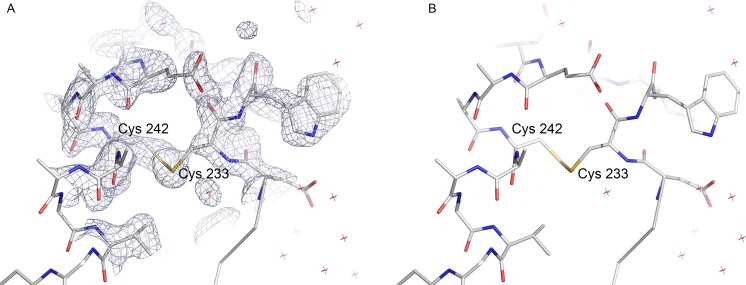
A. Composite omit map contoured at 1σ for the region containing the SS bond in *Sm*MTAP. B. Stick model for the same region showed in A. This disulphide bond is formed by the residues Cys 233 and Cys 242 only in some Apo mutant structures. This SS bond helps maintain the gate loop in open conformation and could be involved in the nucleoside accessibility of the active site.

A disulphide bond between residues C233 and C242 was observed in the APO structures of S12T, N87T and S12T/N87T mutants. *Sm*MTAP structures displaying this SS bond also have the gate loop locked in open form. It remains to be characterized why there is a preference for the formation of such bonds in these mutants and the biological relevance (if any) of this SS bond (Fig 4).

In general, residues 229–242, which comprise the catalytic residue D230 and the gate loop, are disordered in subunits without ligands. Residues 242–253 of the H8 helix in Apo subunits are in different orientations compared to the bound structure.

### Active site description

**Fig 5:**
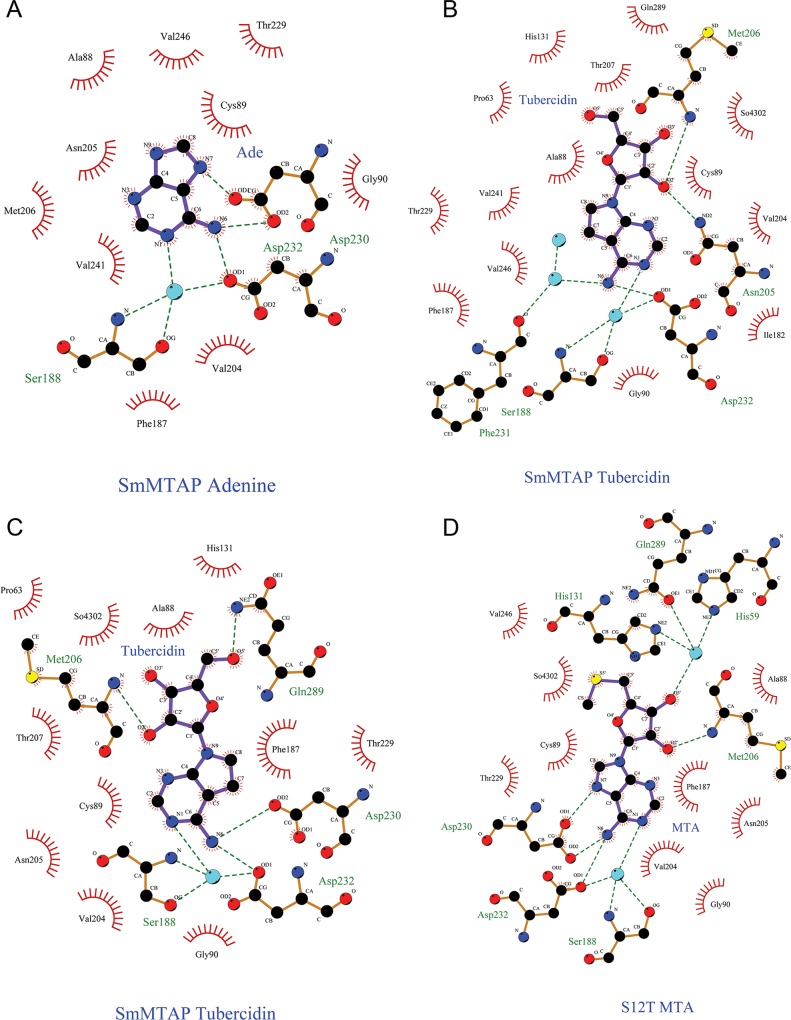
Ligplus^+^ diagrams for ligands in the *Sm*MTAP active site. A. adenine; B. tubercidin; C., different binding modes of tubercidin in the active site interacting with both D230 and Q289 residues. D. MTA.

The active site of *Sm*MTAP was characterized using complexes with adenine, MTA, tubercidin and sulphate. Tubercidin is an adenosine analogue (7-deazaadenosine) that shows potent activity against *S*. *mansoni* and is an inhibitor of adenosine phosphorylase activity [15]. All complexes were obtained from a crystal that was grown in the presence of 5 mM of ligand. Similar to other MTAPs, the active site is located near the interface between monomers. Residues H131 and Q289 from neighboring subunit also participate in the active site. Ligplus^+^ diagrams for all ligands are shown in Fig 5. The active site is subdivided into three sub-sites: base, ribose/methylthioribose and phosphate binding sites, as described for NPs.

### Base Binding Site (BBS)

**Fig 6:**
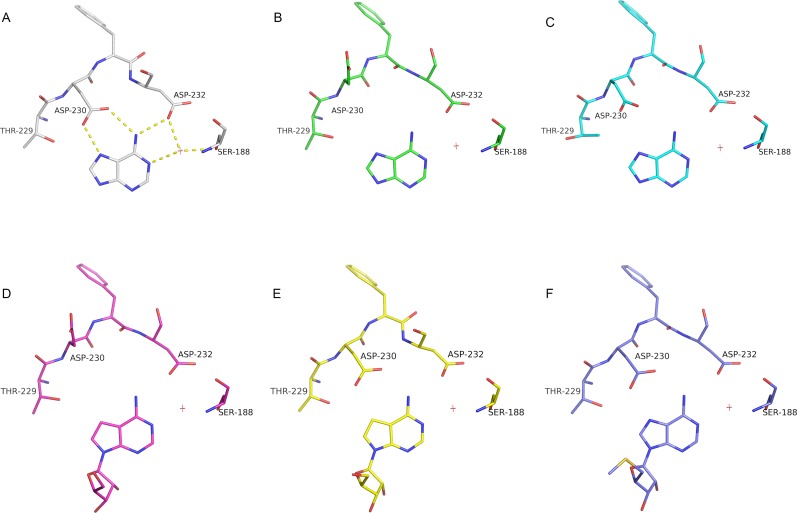
Different binding modes of bases in the main part of BBS residues, showing stick model for residues S118 and 229–232. **A**. H-bond scheme for adenine in the BBS of wild-type *Sm*MTAP. **B**. adenine in S12T/N87T chain B. D230 does not form direct interaction with the base. **C**. In S12T chain C the orientation of D230 is also different. **D**. Tubercidin in wild-type *Sm*MTAP, the side chain of D230 points away to active site. **E**. In Q289L Tubercidin chain A D230 form canonical contacts within the base even being 7-deaza-adenosine. **F**. MTA molecule in S12Tmutant active site. The active site shows high conformational plasticity especially for the side chain of D230 residue.

The adenine molecule or adenine moiety of MTA forms three H-bonds within residues D230 (2) and D232 (1) and one H-bond with a water molecule, which is anchored by the side chain of residues D232 and S118 (Fig 6A). In some structures, adenine does not bind in all subunits; the only WT structure whose BBS are all occupied is the orthorhombic *Sm*MTAP adenine complex. Interestingly, in the S12T adenine complex, the presence of adenine does not cause the gate loop to close (subunits B and C); this is also observed in the S12T/N87T double mutant subunit E (the modes of adenine binding in these structures are shown in Fig 6B and 6C). Despite these exceptions, the MTAP-adenine complex, which is the region near D230, assumes a conformation in which D230 points toward the adenine molecule.

In the MTAP tubercidin complex, all of the active sites are occupied; however, in four of the six subunits, D230 points away from the 7-deazaadenine moiety of tubercidin. Additionally, in subunits that interact within the base (D and E), D230 does not possess canonical contacts with the base; in such cases, different rotamers are observed, and an greater distance between D230 OD1 and C7 of tubercidin is observed compared to the equivalent distance in *Sm*MTAP adenine complex (D230 OD1—N7 adenine). The presence of nitrogen in the N7 position appears to be necessary to orient and maintain the side chain of D230 in the canonical position (Fig 6D). Tubercidin only forms two H-bonds within the BBS, one with D232 (TUB N6—D232 OD1) and one with water 409, which is conserved in all structures with ligand. In the Q289L-tubercidin complex, one BBS is in the canonical D230 conformation (Fig 6D and 6E).

A complex between the *Sm*MTAP mutant S12T and MTA was also obtained; in this case, MTA bound all subunits, causing gate loop closure in the A and B subunits, whereas subunit C was in open conformation. The adenine moiety of the MTA forms the same interactions described above for adenine (Fig 6F). All binding modes in BBS are visualized in Fig 6.

**Fig 7:**
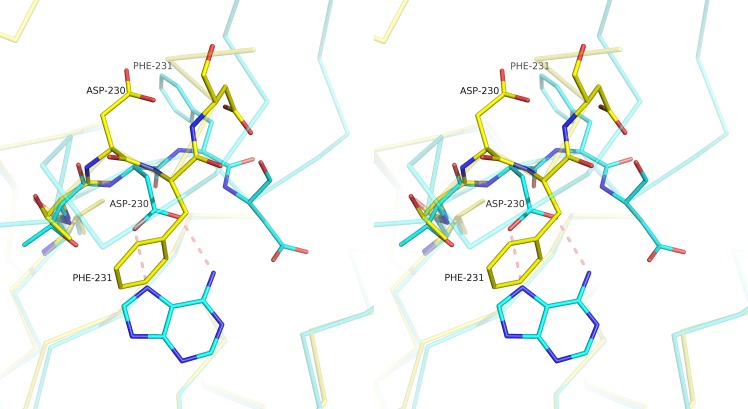
Stereo image for residues 229–232 showing the large movement involved in ligand binding. The *Sm*MTAP adenine complex is shown in cyan, and the Apo form is shown in yellow. In Apo form, D230 points away to the active site, and F231 points towards to the base binding site. This conformational change was not observed in human MTAP and could explain the low K_M_ for adenosine for *Schistosoma* enzyme.

Comparing the BBS between bound and unbound structures reveals a large movement involving residues 230–232 (usually residues 233–241 are absent in the APO structures) (Fig 7), and residue T229 presents a different side chain rotamer. In APO structures, E230 usually points away from the active site, and F231 occupies part of the base binding site (BBS); this movement involves the reorientation of both the main and side chains. The distances between the Cαs of residues 230–232 are 2.1, 6.7 and 6.4 Å, respectively. This movement was not observed for MTAPs from other organisms.

### Methylthioribose/ Ribose Binding Site (RBS)

The characterization of RBS was based on 16 monomers of tubercidin complexes (6 monomers of the wild type and 3 of the Q289L mutant), 3 monomers of MTA (all S12T) and 4 monomers of glycerol (from the WT adenine complex). Ribose/MTR is a mainly hydrophobic binding site; however, four H-bonds were observed in this site.

The RBS is formed by the H-bonded interacting residues N205, M206, D232 and Q289 and the non-bonded interacting residues A88, H131 and V246.

The glycerol that was found in RBS in some structures with adenine came from the cryoprotection solution. The carbon atoms of glycerol lie in the same position of C3', C4' and C5' in the ribose moiety of nucleoside. The glycerol forms four H-bonds: one with residue S12, one with adenine N9 and two with the water molecules present in RBS.

The ribose moiety of tubercidin forms four H-bonds within RBS (O2'—N205 ND2; O2'—M206 N; N6—D232 OD1 and O5'—Q289 OE1). This latter is only possible due to the presence of glutamine in position 289 (leucine 279 in human MTAP); however, this interaction was not observed in all subunits of the tubercidin complex. Indeed, analyzing the superposition of the 66 monomers obtained showed a high conformational plasticity for the side chain of residue Q289.

For MTA in the S12T structure, only one H-bond is formed in RBS (MTA O2'—M206 N). The presence of the methylthio group in position 5 causes a displacement of the side chain of Q289 to accommodate the bulky group. No other significant differences are observed.

### Phosphate Binding Site (PBS)

The PBS is formed by the residues S12, H55, A88, R54 and T207, which form H-bonds with sulphate, and the non-bonded interacting residues G11 and N87.

**Fig 8:**
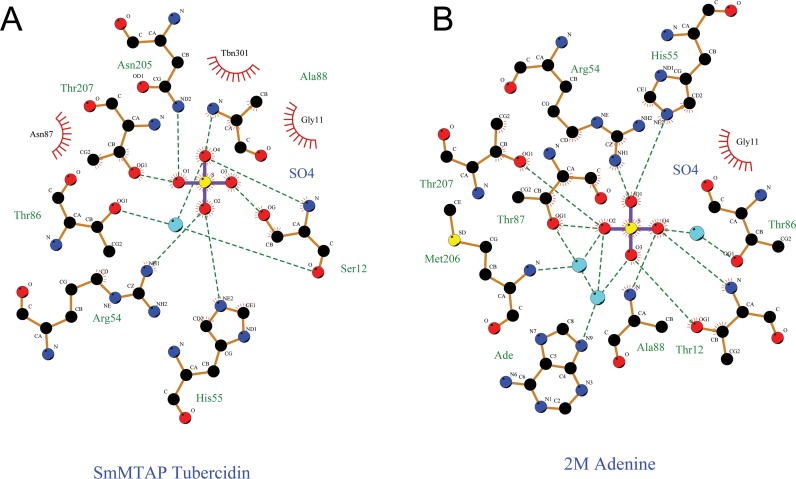
Ligplus^+^ plots for the sulphate molecule. **A**. wt *Sm*MTAP. **B**. Double mutant S12T/N87T and their consequence in sulphate/phosphate binding, where 3 new H-bonds are formed.

The PBS was characterized by the presence of a sulphate molecule. The sulphate molecule forms 7 H-bonds with residues (S12, R54, H55, A88, N205 and T207) in the phosphate binding site (PBS) and one water-mediated bond with T86 (Fig 8).

The orthorhombic *Sm*MTAP-adenine complex does not present sulphate/phosphate molecules in PBS and shows a different conformation for the phosphate loop S12; in this structure, S12 OG interacts with Q289 NE2 via the H-bond (2.88A). It is interesting to note that S12 and Q289 are two sequence substitutions compared to human MTAP. The S12 OG also interacts with glycerol O2 via a weak H-bond.

### Comparison between *Sm*MTAP and human MTAP

When compared by superposition, the human MTAP structures [25,30] do not present larger deviations between structures; however, if we compare *Hs*MTAP and *Sm*MTAP, some differences emerge. The region comprising residues 13–28 shows a different conformation, which appears to be related to the presence of F14 (L20 in *Hs*MTAP) and F250, which reduces the space that S12 loop could assume. In the human structure, residues 23–25 form a 3–10 helix that is not observed in *Sm*MTAP.

Residues 13–28 lie in a low sequence identity region (32% identity). The beta turn between beta strands 8 and 9 presents a different conformation, but there are no structural consequences (this is also observed in the low sequence identity region).

The gate loop (residues 231–242) is also in a different conformation in *Sm*MTAP (when it is present in wt structures) and forms a 3–10 helix (residues 237–239). Although the BBS main residues are fully conserved between *Sm*MTAP and human MTAP, their kinetic properties are entirely different (discussed below). To investigate the structural basis of this kinetic difference, we located three residue substitutions in the active site, S12T, N87T and Q289L (*S*. *mansoni*: human), that could be involved in substrate specificity. Indeed, because the base is the same for adenosine and MTA, these differences are located in both the phosphate and ribose/MTR binding sites.

**Fig 9:**
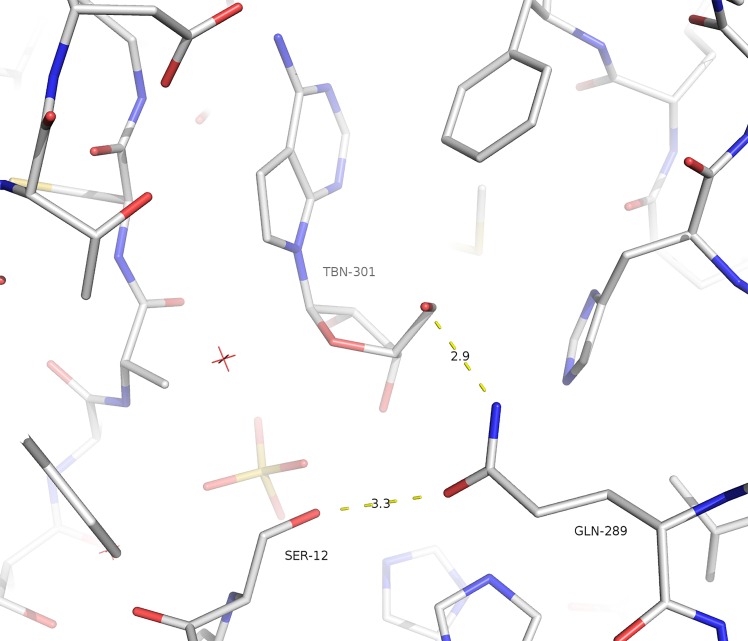
Part of the phosphate and ribose binding sites showing the interactions between the side chain of Q289 and both S12 and O5' of tubercidin. Human enzymes possess leucine in this position and thus do not form these interactions.

The substitution S12T appears to be conservative; however, S12 side chain could assume at least two main conformations: OG pointing towards or away from the PBS. For T18 in human MTAP, only one side chain conformation could interact with phosphate, whereas another consequence of S12 appears to increase the conformational plasticity of the phosphate loop. Serine at this position is observed in other nucleoside phosphorylases as a PNP from human [31] and *S*. *mansoni* [32–38]. As discussed above, the Q289 side chain could interact with S12 in *Sm*MTAP (Fig 9); the consequences of these substitutions are discussed in the section on *Sm*MTAP mutants.

The other substitution, N87T93, is related to the phosphate binding site. In *Hs*MTAP, T93 forms two H-bonds with phosphate by using both OG and main chain nitrogen (as seen in PDB Sum site). In *Sm*MTAP, N87 does not form these interactions within phosphate molecules, although its side chain forms two intra-chain interactions (N87 OD1—S224 OG and N87 ND2—T207 OG1).

The last main difference is the substitution Q289L279. This residue is situated in the last helix (H8) from the other subunit and potentially interacts with the O5' adenosine ribose (as seen in *Sm*MTAP tubercidin complex) and S12. In *Hs*MTAP, the side chain of L279 points towards the hydrophobic MTR binding site. Appleby *et al*. [25] noted that MTAP’s specificity for MTA and other 5'-deoxynuclosides or even for nucleosides with substitutions in 5' position (as halogen, haloalkyl or alkylthio groups) appears to be a consequence of hydrophobicity and lacking H-bond donors or acceptors in the ribose/MTR binding site. The presence of Q289 side chain in ribose/MTR BBS restores the ability to bind adenosine more efficiently. Indeed, in *Sm*MTAP, the tubercidin complex Q298 OE1 forms a weak H-bond (3.19Å) within the O5' of tubercidin, similar to the interaction between H257 and O5' that is observed in human PNP. The side chain of Q289 showed a large conformational plasticity.

### Mutant structures

**Fig 10:**
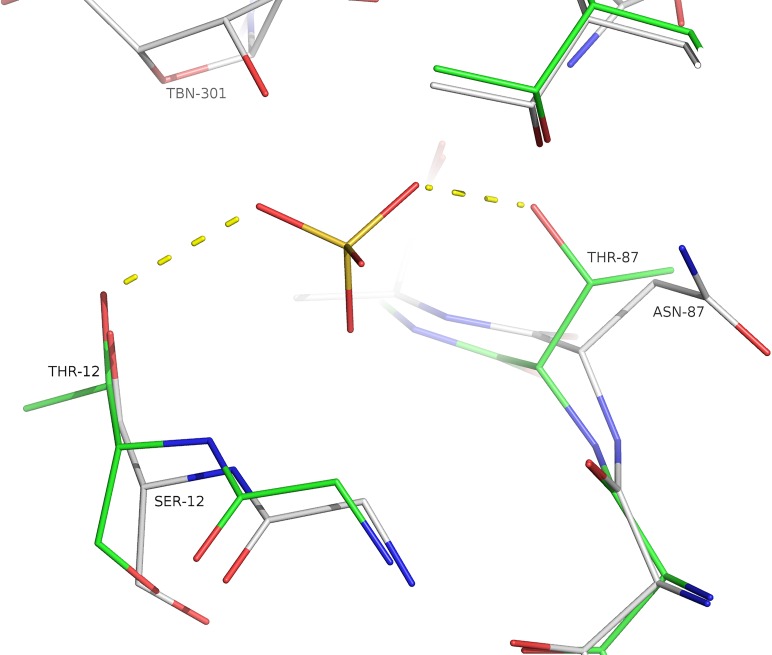
Superposition of wild-type *Sm*MTAP (white) and double mutant S12T/N887T showing the consequence of these mutations in phosphate binding. The side chain of T87 allows an extra H-bond with the phosphate (sulphate), and the presence of T12 only permits one side chain conformation for phosphate interaction; intriguingly, the presence of these mutations increased the phosphate K_M_.

The presence of the mutation S12T does not alter the phosphate loop compared to the *Sm*MTAP WT structures. The main and side chains of T12 forms the same H-bonds with sulphate molecules observed in human MTAP (Fig 10).

The mutant N87T also does not present any large structural differences in comparison to the wt *Sm*MTAP structures; as discussed above, the consequence of this mutation is that the capacity to form a H-bond with phosphate/sulphate in PBS is restored (Fig 10). An interesting consequence of the N87T mutation is the presence of two water molecules near the atoms OD1 and ND2 of T87 mutant occupying approximately the same position of N87 side chain. The bulkier group of N87 prevents the binding of water molecules at these sites. The human MTAP shows equivalent water molecules in these positions. One water molecule interacts with S244 and Y222, and the other interacts with the R54 and T207 side chains; these residues are all conserved in human MTAP. The water interactions with T207 and S224 are the same and are formed by the N87 side chain, thus indicating a necessary conservation of these interactions, even losing the interaction with the phosphate molecule due to the N87 substitution.

The mutant Q289L was prepared to investigate the consequence of the lost H-bond between Q289 side chain and the nucleoside observed in tubercidin *Sm*MTAP complex. The Q289L tubercidin complex permits a direct comparison between structures. A small but consistent difference was observed between Q289L and the WT *Sm*MTAP tubercidin complex, particularly in chain A: an increase in movement at the end of Helix 9 in the Q289L mutants. The L289 permits a larger movement in the end of the helix compared to Q289; when comparing these structures with human MTAP, a larger difference was also observed for residues 277–281 (human MTAP numbering).

We also could obtain the structure of the double mutant S12T/N87T in the Apo form and in complex with adenine. No significant differences were observed between double and single mutants. All of the small differences were discussed above in single mutant structures. Even in the double mutant structures, the position of sulphate/phosphate was conserved. The consequence of these mutations are explained in the kinetics section below.

### Kinetic activity of *Sm*MTAP and mutants

Using a coupled assay with xanthine oxidase (12) we determined the kinetic parameters for MTA, adenosine, PO_4_ and 2'-deoxyadenosine. All measurements were made in sextuplicate in at least three different preparations. The results are shown in Table 2.

**Table 2:** Kinetic parameters for wt *Sm*MTAP and their mutants for adenosine, MTA, 2'-deoxyadenosine, and for adenosine hydrolysis.

		**K**_**M**_ **(μM)**	**k**_**cat**_ **(s**^**-1**^**)**	**V**_**max**_ **(nM/min)**	**k**_**cat**_**/K**_**M**_ **(s**^**-1**^**/ μM**^**-1**^**)**
**Adenosine**	**Wild Type**	**3.14 ± 0.16**	**334.1 ± 6.84**	**50.11**	**106.40 ± 5.84**
	**S12T**	**2.1 ± 0.13**	**98.42 ± 2.36**	**36.91**	**46.86 + 3.11**
	**N87T**	**3.3 ± 0.21**	**62.63 ± 1.51**	**23.49**	**18.97 + 1.29**
	**Q289L**	**1.81 ± 0.08**	**95.79 ± 1.69**	**35.92**	**54.58 ± 2.59**
	**2M**	**3.68 ± 0.14**	**308.9 ± 4.48**	**30.89**	**83.94 ± 3.42**
	**3M**	**7.35 ± 0.36**	**280.3 ± 5.09**	**28.03**	**38.13 ± 1.99**
		**K**_**M**_ **(μM)**	**k**_**cat**_ **(s**^**-1**^**)**	**V**_**max**_ **(nM/min)**	**k**_**cat**_**/K**_**M**_ **(s**^**-1**^**/ μM**^**-1**^**)**
**MTA**	**Wild Type**	**3.63 ± 0.14**	**443.8 ± 5.72**	**66.57**	**122.25 ± 4.97**
	**S12T**	**1.22 ± 0.09**	**145.1 ± 4.53**	**54.4**	**118.93 ± 9.53**
	**N87T**	**0.71 ± 0.04**	**84.62 ± 2.24**	**31.73**	**119.18 ± 7.42**
	**Q289L**	**2.49 ± 0.1**	**241.6 ± 3.91**	**72.48**	**97.02 ± 4.20**
	**2M**	**2.12 ± 0.08**	**416.2 ± 7.20**	**41.62**	**196.32 ± 8.15**
	**3M**	**3.12 ± 0.12**	**253.9 ± 3.61**	**25.39**	**81.37 ± 3.34**
		**K**_**M**_ **(μM)**	**k**_**cat**_ **(s**^**-1**^**)**	**V**_**max**_ **(nM/min)**	**k**_**cat**_**/K**_**M**_ **(s**^**-1**^**/ μM**^**-1**^**)**
**2'deoxy adenosine**	**Wild Type**	**3.97 ± 0.41**	**224.3 ± 11.31**	**84.13**	**56.49 ± 6.49**
	**S12T**	**0.99 ± 0.09**	**48.44 ± 1.40**	**18.18**	**48.92 ±4.67**
	**N87T**	**10.33 ± 0.78**	**441.2 ± 16.23**	**41.04**	**42.71 3.59**
	**Q289L**	**3.02 ± 0.23**	**80.03 ± 2.28**	**30.01**	**26.5 ±2.15**
	**2M**	**3.89 ± 0.16**	**372.8 ± 6.31**	**37.28**	**95.83 ± 4.26**
	**3M**	**6.52 ± 0.32**	**299.2 ± 5.28**	**29.92**	**45.88 ± 2.39**
		**K**_**M**_ **(μM)**	**k**_**cat**_ **(s**^**-1**^**)**	**V**_**max**_ **(nM/min)**	**k**_**cat**_**/K**_**M**_ **(s**^**-1**^**/ μM**^**-1**^**)**
**PO**_**4**_	**Wild Type**	**65.58 ± 4.69**	**477.6 ± 7.47**	**35.82**	**7.28 ± 0.53**
	**S12T**	**10370 ± 998**	**116.1 ± 5.30**	**43.54**	**0.011 ± 0.00**
	**N87T**	**56.1 ± 6.64**	**392.4 ± 8.22**	**147.2**	**6.99 ± 0.84**
	**Q289L**	**288.1 ± 26.07**	**50.32 ± 1.89**	**18.87**	**0.17 ± 0.02**
	**2M**	**142.6 ± 7.15**	**524.2 ± 8.21**	**52.42**	**3.67 ± 0.19**
	**3M**	**434 ± 41,0**	**12,79 ± 0,38**	**1,27**	**0,029 ± 0,093**
		**K**_**M**_ **(μM)**	**k**_**cat**_ **(s**^**-1**^**)**	**V**_**max**_ **(nM/min)**	**k**_**cat**_**/K**_**M**_ **(s**^**-1**^**/ μM**^**-1**^**)**
**Adenosine w/o PO**_**4**_	**Wild Type**	**4.6 ± 0.39**	**175.9 ± 3.84**	**16.36**	**38.23 ± 3.35**
	**S12T**	**No reaction**			
	**N87T**	**8.86 ± 0.52**	**616.2 ± 12.64**	**57.31**	**69.54 ± 4.32**
	**Q289L**	**No reaction**			
	**2M**	**38.74 ± 4.19**	**160.5 ± 8.34**	**16.05**	**4.14 ± 0.5**
	**3M**	**17.39 ± 1.36**	**131.9 ± 4.02**	**13.19**	**7.58 ± 0.64**

The *Sm*MTAP has a lower K_M_ for adenosine (3.14 ± 0.16 μM), 2'-deoxyadenosine (3.97 ± 0.41 μM) and MTA (3.63 ± 0.14 μM), as observed for other parasites (11). The K_M_ for PO_4_ was also obtained (65.58 ± 4.69 μM). These data show that *Sm*MTAP is well suited for both substrates (adenosine and MTA), in clear opposition to the human MTAP, which displays a higher affinity for MTA as substrate compared to adenosine (K_M_ = 1.8 and 184 μM, respectively).

The parasite *Trypanosoma brucei* has an MTAP with low K_M_ for MTA (2 μM) and adenosine (21 μM). Searching the Brenda database, we failed to find an MTAP with a lower K_M_ for adenosine and 2'-deoxy adenosine than we found for *Sm*MTAP. Therefore, we could assume that *Sm*MTAP is the adenosine phosphorylase discovered by Miech *et al*. [2] using *S*. *mansoni* extracts.

Despite the low K_M_ for the MTA, adenosine and 2'-deoxyadenosine, the *Sm*MTAP has high k_cat_ values (Table 2) and is 7.1 times greater for MTA compared to the human enzyme [39]; unfortunately, we could not perform a direct comparison for adenosine k_cat_ due to the lack of literature data. The k_cat_ values for adenosine and 2'-deoxyadenosine are 75.2 and 50.5%, respectively; the MTA k_cat_ values indicates a slight catalytic preference for MTA. This is also reflected in the k_cat_/K_M_ relationship.

As discussed above, we chose three residues in the RBS and PBS for site-directed mutagenesis (S12, N87 and Q289) to investigate their roles in the kinetic parameters of MTA, adenosine, 2-deoxyadenosine and PO_4_. We also generated double S12T/N87T and triple mutants S12T/N87T/Q289L.

Interestingly, these mutants all reduced the K_M_ for MTA with the S12T and N87T, showing decreases of approximately 3- and 5-fold. A reduction in k_cat_ was observed for S12T and N87T (3- and 5-fold decrease). The double mutant appears to be conservative with respect to these parameters and shows a small increase in the k_cat_/K_M_ parameter (1.6X), thus indicating a conservative role of this pair of residues in MTA catalysis.

A different scenario was observed for adenosine. The triple mutant increased the K_M_ value 2.3-fold, whereas other mutants did not have great effects on K_M_. The double and triple mutants have conservative k_cat_ values; however, S12T, N87T and Q289L reduce the k_cat_ value by 3.4-, 5.3- and 3.4- fold, respectively. Nonetheless, a great difference was observed in the k_cat_/K_M_ values with respect to the wild-type enzyme. All mutants affected this parameter, reducing it by 5.5-fold (N87T), ~ 2-fold (S12T, Q289L) and 2.7-fold for the triple mutant.

When 2'-deoxyadenosine was used as substrate, a similar decrease in K_M_ was observed for S12T (in comparison to adenosine); however, the N87T mutant showed a 2.6X increase in K_M_ in relation to the wt *Sm*MTAP with adenosine. This increase was not observed in the double mutant. The k_cat_ was significantly altered for S12T (4.6X decrease), N87T (2X increase), and Q298L (2.8X decrease), and both double and triple mutants showed increased k_cat_ values (1.6X and 1.3X, respectively). The k_cat_/K_M_ value did not exhibit significant differences in S12T, N87T and triple mutants; the Q289L showed a 2-fold decrease.

These mutations were all unable to revert the adenosine catalysis by *Sm*MTAP when using the corresponding human MTAP residues, even the Q289L mutant, the side chain of which could form an H-bond with the ribose moiety of adenosine. However, the triple mutant showed an increase in K_M_ and a decrease of catalytic efficiency for adenosine. This could be explained by the fact that the residues that interact with the adenine moiety in BBS are fully conserved between human and schistosome enzymes. Therefore, the efficient catalysis of adenosine is a result of other substitutions in the *S*. *mansoni* enzyme and/or the larger movements of the gate loop (which is not observed in human MTAP). This is in line with Hammes [40], who provided a holistic vision of enzyme structures, where the net of H-bonds within the structure is responsible for and collaborate to realize catalysis.

As discussed above, all of the planned mutations were performed in both RBS and PBS; the *Sm*MTAP has a lower K_M_ for PO_4_ compared to the human enzyme (65.58 versus 320 μM), and we successfully reverted the K_M_ to the value for the human MTAP in both Q289L and the triple mutant. These mutants showed an increase in K_M_ of 4.4X and 4.1X, respectively. Intriguingly, the results show an increase of 158X in the PO_4_ K_M_ for the S12T mutant. In contrast, the N87T mutant shows K_M_ values near the wt *Sm*MTAP, thus reflecting the consequence of an extra H-bond within the PO_4_ site and could thus compensate for the presence of T12 in double and triple mutants. The k_cat_/K_M_ was also significantly reduced by 43X (Q289L), 303X (triple mutant) and ~2X (double mutant), thus indicating the importance of Q289 in the catalysis and possibly reflecting the importance of reduced flexibility at the end of Helix 9.

### *Sm*MTAP could cleave adenosine in phosphate absence

An intriguing observation was the capacity of wt *Sm*MTAP to catalyze adenosine cleavage in the absence of phosphate. In this case, *Sm*MTAP was extensively dialyzed against Hepes buffer prior to its utilization for kinetic experiments. We were not able to obtain kinetic data for hydrolysis with MTA. The S12T and Q289L mutants did not provide reliable data either. The wt *Sm*MTAP K_M_ for adenosine in the absence of phosphate is 4.6 μM, which is 1.4X higher than that obtained in the presence of phosphate; however, the k_cat_ is 2-fold lower, and the k_cat_/K_M_ is reduced 2.78-fold. The N87T mutant shows a 2.8-fold increase in K_M_, an increase in k_cat_ (1.8X) and a decrease in k_cat_/K_M_ (1.53X). In the double mutant, the K_M_ is increased approximately 12.3X, k_cat_ is reduced 2-fold and k_cat_/K_M_ is 25.7-fold lower than in the presence of phosphate. The triple mutant resembles the double mutant, with a K_M_ and k_cat_ that are 5.5X and 2.5 lower, respectively and a 14-fold reduction in the k_cat_/K_M_ relationship. Interestingly, both double and triple mutants could restore adenosine cleavage in the absence of phosphate; indeed, the Q289L and 3M mutants show higher K_M_ values for phosphate and low k_cat_/K_M_, thus demonstrating its great impact on the K_M_ and catalytic efficiency of these mutants. Catalysis of nucleosides by nucleoside phosphorylases in the absence of phosphate was observed for bovine uridine phosphorylase, where the enzyme produces a free base and a glycal as products of catalysis [41], and for calf spleen purine nucleoside phosphorylase [42,43]. This could be the reason for our failure to obtain adenosine or MTA complexes for wt *Sm*MTAP, for which we only observed adenine in the active site, even when using ammonium sulphate in crystallization conditions.

## Discussion

### Metabolic context of *Sm*MTAP/AP

In humans, the MTAP enzyme functions solely in the polyamine pathway by removing MTA produced by spermidine and spermine synthases. Thus, MTAP phosphorolysis is the only way to metabolize MTA in humans and, more generally, in mammals [16].

MTA is produced in the polyamine pathway, where two MTA molecules are produced via the conversion of putrescine into spermine. The MTA phosphorolysis by MTAP produces adenine and 5’-methythioribose-1-phosphate (MTR1P). The adenine base is salvaged by the action of APRT, and MTR1P is converted into methionine by the methionine salvage pathway (MSP).

One advantage of the MSP may be the cost of sulfur assimilation; therefore, the MSP keeps the sulfur inside the cell, especially for species that live in sulfur-restricted environments [44]. In contrast, species living in sulfur-abundant environments lack MTR kinases and the MSP. The same study reported an advantage for possessing a nucleosidase: the adenine could be used as a purine source.

*S*. *mansoni* genome analysis using either GeneDB [45] and KEGG reveals that the parasite lacks all enzymes of a polyamine pathway: ornithine decarboxylase (E.C 4.1.1.17), spermidine synthase (E.C 2.5.1.16), and spermine synthase (E.C 2.5.1.22), which produces MTA. Another intriguing fact is the destination of MTR1P in the methionine salvage pathway. This pathway is also completely absent in *S*. *mansoni;* indeed, there is no coding sequence for any of the enzymes involved in the transformation of MTR1P into methionine: methylthioribose-1-phospate isomerase (E.C 5.3.1.23), methylthioribulose-1-phospate dehydratase (E.C 4.2.1.109), Enolase-phosphatase E1 (E.C. 3.1.3.77), Acireductone dioxygenase 1 (E.C 1.13.11.54) and aromatic-amino-acid transaminase (E.C 2.6.1.57).

In higher eukaryotes, methionine is an essential amino acid that is required in diet supplementation and is one of the limiting amino acids. However, this is not the scenario for *S*. *mansoni* because the parasite lives in blood; therefore, methionine is not difficult to obtain because the principal source of amino acids is the human blood, where hemoglobin is found in endless stock for the parasite. Human hemoglobin has 1.7% of methionine in its composition.

Certainly, host-parasite interactions generate selective pressure, which drives the redrawing of metabolic pathways and incurs the loss of a significant number of genes associated with biosynthetic functions because the parasite depends on the host to obtain its supply of metabolites and precursors [46]. This is true for the purine, sterols and fatty acid "*de novo*" pathways in *S*. *mansoni* [4,47,48].

Payne and Loomis [49] analyzed the complete genome of protists, Dictyostelium and six animals for the retention and loss of the biosynthetic amino acids pathways and concluded “when an organism becomes a consumer by eating other organisms, all of the amino-acids are available in the diet and no longer need to be synthesized”. If these pathways are not used in other essential functions, they are useless and dispensable. Genes in these nonessential pathways accumulate deleterious mutations, become non-functional and are eventually deleted from the genome.

The absence of polyamine and methionine salvage pathways, the high activity of adenosine phosphorolysis, and the low K_M_ and high k_cat_ for adenosine make the *Sm*MTAP an exclusive component of the purine salvage pathway. Thus, it is an adenosine phosphorylase, as described by Senft and collaborators more than 30 years ago.

Recently, Savarese and El Kouni [50] isolated two adenosine phosphorylases from *S*. *mansoni* extracts: the first one cleaves MTA, adenosine and 2’-deoxyadenosine to adenine and their respective sugar phosphates, and the second utilizes guanosine, inosine, adenosine and 2’ deoxyadenosine, the last in clear contrast to our observations of *S*. *mansoni* PNP, for which adenosine is a weak inhibitor [38]. However, the MTAP enzyme described by Savarese does not present hydrolytic activity against MTA, as we observed for recombinant *Sm*MTAP; however, a very low hydrolytic activity (in the error range of measurements) was observed for adenosine. Thus, we could assume that the first extract is the MTAP described here due to their similar activity characteristics and that the latter is another uncharacterized PNP isoform due to its guanosine/inosine/adenosine cleavage capacity. However, a direct comparison is impossible due to the absence of SDS-PAGE for both fractions (to evaluate the monomer molecular weight (MW)), analytical gel filtration and/or Dynamic Light Scattering (DLS) to estimate the MW of these enzymes in solution.

The discovery of MTAP/AP properties from *S*. *mansoni* confirms the findings of Senft and co-workers, who described an activity of adenosine phosphorylase that was distinct from that of the purine nucleoside phosphorylase enzyme. Truly, our previous finding that adenosine is a weak inhibitor of *Sm*PNP activity discards PNP as being responsible for this activity [38].

The *S*. *mansoni* genome data demonstrate a complete absence of the methionine salvage and polyamine pathways and suggest a different role of *Sm*MTAP in parasite metabolism. Therefore, we can assume that *Sm*MTAP/AP is the sole protein responsible for the conversion of adenosine (and, to a lesser extent, MTA) into adenine, which is subsequently converted into AMP by APRT (the metabolic branch responsible for 70% of the conversion of the incoming adenosine into AMP).

Site directed mutagenesis experiments could not revert the adenosine phosphorolysis phenotype due to fully conserved BBS residues; thus, other residues that are not directly involved in catalysis could be responsible for this activity. The gate loop, which undergoes large conformational changes between APO and ligand structures, could be involved, in contrast to the lack of conformational plasticity observed for human MTAP.

The high adenosine phosphorolysis activity, in contrast to that observed in human MTAP, probably evolved due to selective pressure from the abundance of adenosine in human serum, which forced the parasite to be more adapted to utilize adenosine. This finding is in line with the absence of MTA producer enzymes and destination enzymes, which could handle the MTR1P product of MTA phosphorolysis.

An intriguing open question is about the role of *Sm*MTAP in the *S*. *mansoni* vomitus [51], where the enzyme could be responsible for the conversion of adenosine into adenine, with the subsequent incorporation of adenine into the worm nucleotide pool. The presence of adenosine phosphorylase activity in Fisher medium after worm removal was observed by Sent *et al*. [7]. This finding is in line with the adenine incorporation in *S*. *mansoni*, which was 6 to 10 times higher than observed for mammalian cell lines [6].

This *Sm*MTAP structure corroborates our efforts to solve all of the structures of the enzymes that are involved in purine salvage pathway; indeed, this is the fourth different structure solved by our group, following purine nucleoside phosphorylase [32–38], adenosine kinase [52], and adenylate kinase [53]. We hope that this effort increases the structural basis of purine metabolism in *S*. *mansoni* and, together with kinetic data, will aid in the development of new alternative treatments for this important but neglected disease, for which *Sm*MTAP is described as a potentially chemotherapeutic target.

## Supporting Information

S1 Fig Adenosine phosphorolysis. (TIF)

S2 Fig MTA phosphorolysis (TIF)
