# Supplementary figures and images for: Crystal Structure of Schistosoma mansoni Adenosine Phosphorylase/5’-Methylthioadenosine Phosphorylase and Its Importance on Adenosine Salvage Pathway
Source: PLoS Negl Trop Dis. 2016 Dec 9;10(12):e0005178. doi: 10.1371/journal.pntd.0005178 (PMC5147791; doi:10.1371/journal.pntd.0005178)

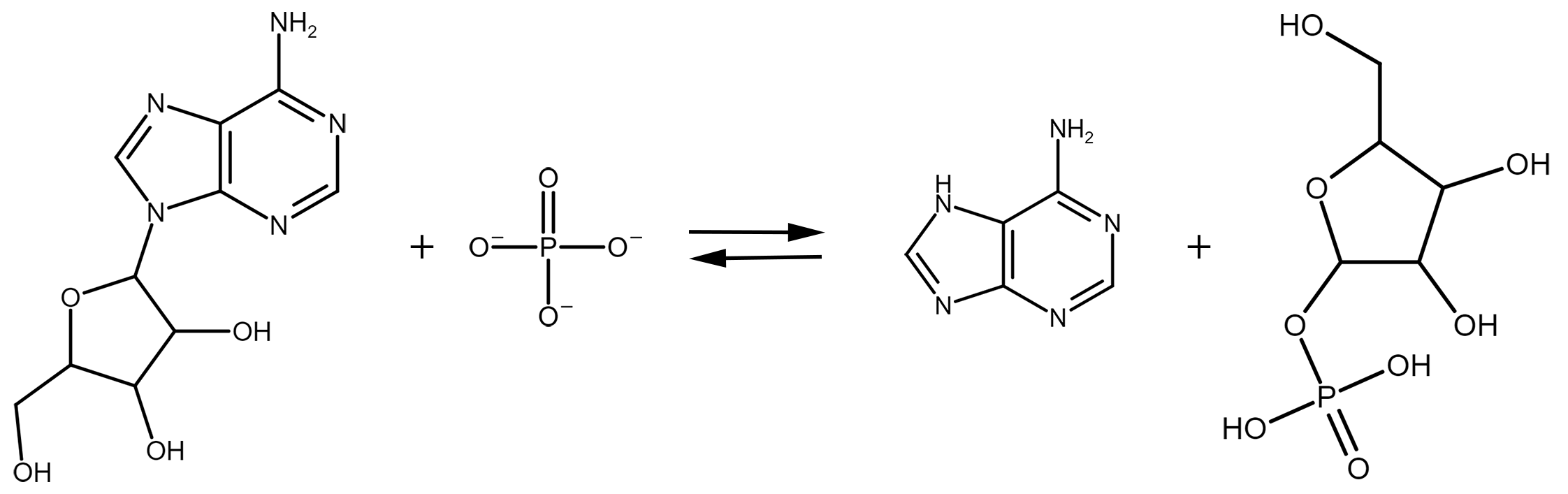

Supplement: S1 Fig — (TIF) [file pntd.0005178.s001.tif]

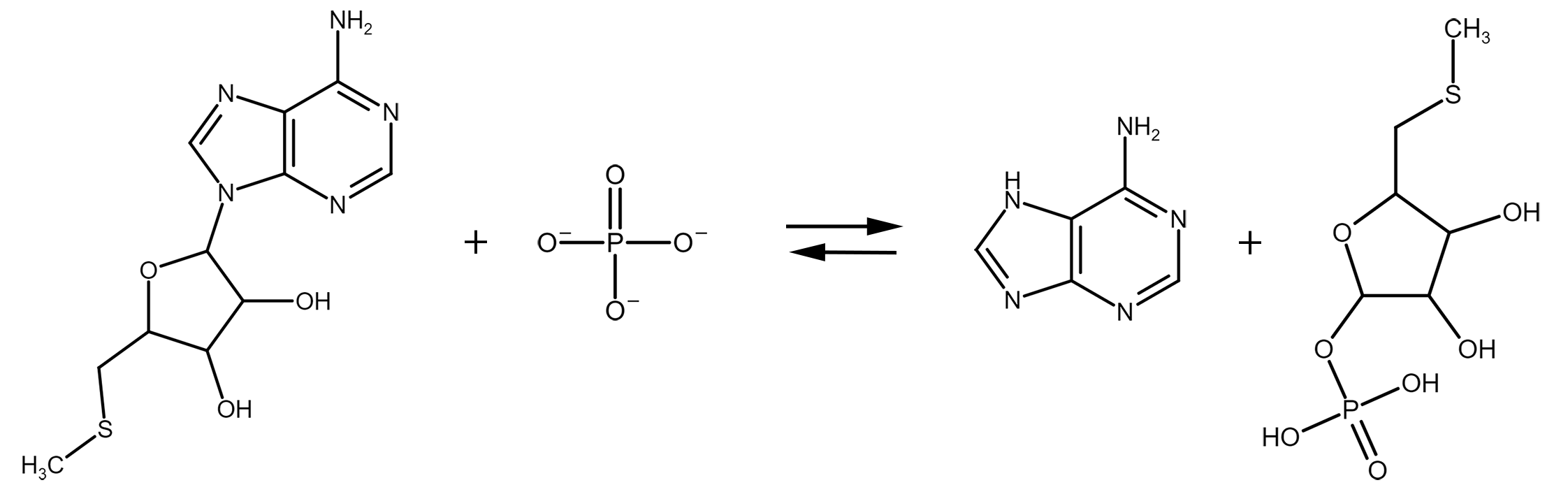

Supplement: S2 Fig — (TIF) [file pntd.0005178.s002.tif]
